# A Universal DNA Aptamer that Recognizes Spike Proteins of Diverse SARS‐CoV‐2 Variants of Concern

**DOI:** 10.1002/chem.202200078

**Published:** 2022-02-18

**Authors:** Zijie Zhang, Jiuxing Li, Jimmy Gu, Ryan Amini, Hannah D. Stacey, Jann C. Ang, Dawn White, Carlos D. M. Filipe, Karen Mossman, Matthew S. Miller, Bruno J. Salena, Deborah Yamamura, Payel Sen, Leyla Soleymani, John D. Brennan, Yingfu Li

**Affiliations:** ^1^ Department of Biochemistry and Biomedical Sciences McMaster University 1280 Main Street West Hamilton, Ontario L8S 4K1 Canada; ^2^ Biointerfaces Institute McMaster University 1280 Main Street West Hamilton, Ontario L8S 4K1 Canada; ^3^ Michael G. DeGroote Institute of Infectious Disease Research McMaster University 1280 Main Street West Hamilton, Ontario L8S 4K1 Canada; ^4^ McMaster Immunology Research Centre McMaster University 1280 Main Street West Hamilton, Ontario L8S 4K1 Canada; ^5^ Department of Chemical Engineering McMaster University 1280 Main Street West Hamilton, Ontario L8S 4K1 Canada; ^6^ Department of Medicine McMaster University 1280 Main Street West Hamilton, Ontario L8S 4K1 Canada; ^7^ Department of Pathology and Molecular Medicine McMaster University 1280 Main Street West Hamilton, Ontario L8S 4K1 Canada; ^8^ School of Biomedical Engineering McMaster University 1280 Main Street West Hamilton, Ontario Canada L8S 4K1

**Keywords:** aptamer, COVID-19, diagnostics, spike protein, variants of concern

## Abstract

We report on a unique DNA aptamer, denoted MSA52, that displays universally high affinity for the spike proteins of wildtype SARS‐CoV‐2 as well as the Alpha, Beta, Gamma, Epsilon, Kappa, Delta and Omicron variants. Using an aptamer pool produced from round 13 of selection against the S1 domain of the wildtype spike protein, we carried out one‐round SELEX experiments using five different trimeric spike proteins from variants, followed by high‐throughput sequencing and sequence alignment analysis of aptamers that formed complexes with all proteins. A previously unidentified aptamer, MSA52, showed *K*_d_ values ranging from 2 to 10 nM for all variant spike proteins, and also bound similarly to variants not present in the reselection experiments. This aptamer also recognized pseudotyped lentiviruses (PL) expressing eight different spike proteins of SARS‐CoV‐2 with *K*_d_ values between 20 and 50 pM, and was integrated into a simple colorimetric assay for detection of multiple PL variants. This discovery provides evidence that aptamers can be generated with high affinity to multiple variants of a single protein, including emerging variants, making it well‐suited for molecular recognition of rapidly evolving targets such as those found in SARS‐CoV‐2.

## Graphical abstract

**This is a unique one**: A unique DNA aptamer has been discovered that has the ability to recognize the spike proteins of the wildtype SARS‐CoV‐2 and its seven current variants of concern (VoCs), including B.1.1.7, B.1.351, P.1, B.1.429, B.1.617.1 B.1.617.2 and B.1.1.529 and is predicted to recognize the spike proteins of future VoCs.

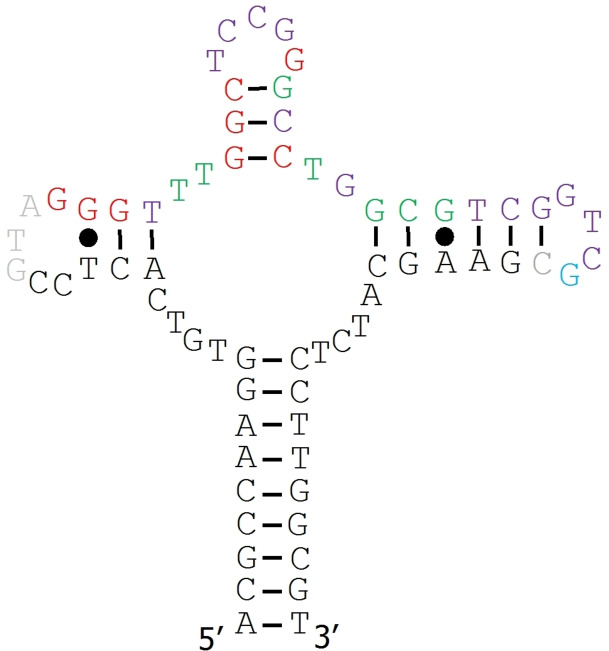

The COVID‐19 pandemic, caused by severe acute respiratory syndrome coronavirus 2 (SARS‐CoV‐2), has become a huge burden to our society.^[1]^ The initial virus was discovered in December, 2019 in the city of Wuhan, China, then spread globally. Since then, several variants of concern (VoCs) have emerged, the most notable VoCs being B.1.1.7 (the UK variant; Alpha), B.1.351 (the South Africa variant; Beta), P.1 (the Brazil variant; Gamma), B.1.429 (the California variant; Epsilon), B.1.617.2 (the Indian variant; Delta),^[2]^ and very recently B.1.1.529 (Omicron).^[3]^

Large‐scale testing, contact tracing and isolation have been implemented as methods to control the spread of SARS‐CoV‐2. However, current testing using quantitative reverse‐transcription real‐time polymerase chain reaction (qRT‐PCR), while highly sensitive and specific for detecting SARS‐CoV‐2, suffers from a slow turnaround time and high cost, making it unsuitable as a screening tool to enable rapid responses to outbreaks.^[4]^ Several commercial Antigen (Ag) tests, such as the Abbott PanBio^TM^ COVID‐19 Ag test, the Abbott BINAX Now^TM^ COVID‐19 Ag test, and the Ellume rapid COVID‐19 test, are relatively rapid and inexpensive, but are inherently less sensitive than RT‐PCR assays.[^5^, ^6^] In addition, rapid tests such as the Quidel QuickVue At‐Home OTC COVID‐19 Test and Abbott BinaxNOW COVID‐19 Antigen Self‐Test, have been reported to be insufficiently sensitive to detect the Omicron variant until several days after becoming infectious, showing that rapid tests are unable to detect emerging variants as well as previous variants.^[7]^ With constant mutations of the genome of SARS‐CoV‐2 and rapid emergence of new VoCs, there is an urgent need for simpler, faster, more cost‐effective large‐scale testing methods that work with both current and emerging VoCs.

DNA aptamers, which can be selected from random sequence pools by in vitro selection (SELEX),[^8^, ^9^] offer several key advantages over antibodies for the development of rapid tests, such as small size, high chemical and thermal stability, easy and precise modification, scalable production and minimal batch‐to‐batch variation.^[10]^ In addition, they have been shown to be capable of detecting targets present in clinical samples.[^11^, ^12^, ^13^] These attractive properties make aptamers important MREs for assay and diagnostic development.[^14^, ^15^, ^16^, ^17^]

Several groups including ours have isolated DNA aptamers that bind the S1 subunit of the spike protein of the original SARS‐CoV‐2 virus or its receptor‐binding domain (RBD).[^18^, ^19^, ^20^, ^21^, ^22^, ^23^, ^24^, ^25^, ^26^, ^27^] However, other than our finding that two of our S1‐binding DNA aptamers, named MSA1 and MSA5, also exhibited similar affinity for the spike proteins of the B.1.1.7 variant of concern, no information is available on whether the reported aptamers can bind the spike proteins of other important VoCs.^[27]^

As the starting point for examining our aptamers for recognition of variant spike proteins, we used dot blot assays (Figure S1) to examine the binding affinity of MSA1 (Figure 1A) and MSA5 (Figure 1B) for the full trimeric spike proteins of the original Wuhan SARS‐CoV‐2 (WHS) and four VoCs: B.1.1.7S (UKS), B.1.429S (CAS), B.1.351S (SAS) and P.1S (BZS; “S” in the names stands for the full spike protein). Note that at the time of these experiments, neither the S1 nor full trimeric spike proteins were available for the Delta or Omicron variants. MSA1 showed strong affinity for both B.1.1.7S (*K*_d_=1.2 nM) and B.1.429S (*K*_d_=1.3 nM), but had significantly reduced affinity for P.1S (*K*_d_=75 nM) and very poor affinity for B.1.351S (*K*_d_>200 nM), as compared to the *K*_d_ of 19.8 nM for the original Wuhan virus. MSA5 showed similar levels of affinity for WHS (*K*_d_=5.6 nM), B.1.1.7S (*K*_d_=4.2 nM), B.1.429S (*K*_d_=5.0 nM), and B.1.351S (*K*_d_=8.2 nM). However, it had significantly decreased affinity for P.1S (*K*_d_=52 nM).

**Figure 1:**
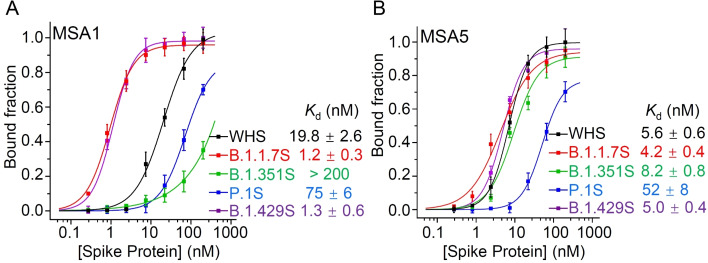
Assessment of binding affinity of A) MSA1 and B) MSA5 for the spike protein of the original SARS‐CoV‐2 (WHS) and four variant spike proteins (B.1.1.7S, B.1.351S, P.1S, B.1.429S) using dot blot assays.

The results presented above suggest that the epitopes for MSA1 and MSA5 are not identical, and more importantly, the epitopes recognized by both aptamers are sensitive to the conformational changes of the spike proteins caused by the mutations associated with some of the VoCs. Specifically, MSA5 is sensitive to the conformational changes of P.1S while MSA1 is sensitive to that of both P.1S and B.1.351S. We also examined six other published aptamers (randomly chosen) for binding to the spike proteins and none of them were able to recognize WHS, B.1.1.7S, B.1.351S and P.1S with uniformly excellent affinity (Table S1). These findings suggest that these aptamers cannot function as universal affinity agents targeting all variant spike proteins for either therapeutic or diagnostic applications.

Our recently published aptamers for SARS‐CoV‐2 were selected using a pre‐structured DNA library that placed a 40‐nt random region (Figure 2A) in a hairpin‐structured arrangement often observed with many published aptamers, resulting in the isolation of MSA1 and MSA5, the top ranked and 5^th^ ranked aptamer candidates.^[27]^ However, further examination of the binding affinity of other aptamers, including two low‐ranked aptamers, MSA50 (the 50^th^ ranked aptamer candidate; *K*_d_=10.2 nM) and MSA439 (the 439^th^ ranked candidate; *K*_d_=36.9 nM), showed that even lowly ranked candidates in the final pool still exhibited excellent affinity for WHS. Based on these results, we hypothesized that our enriched aptamer pool might also contain aptamer candidates that could universally recognize the spike proteins of the currently circulating VoCs and perhaps even emerging VoCs.

**Figure 2:**
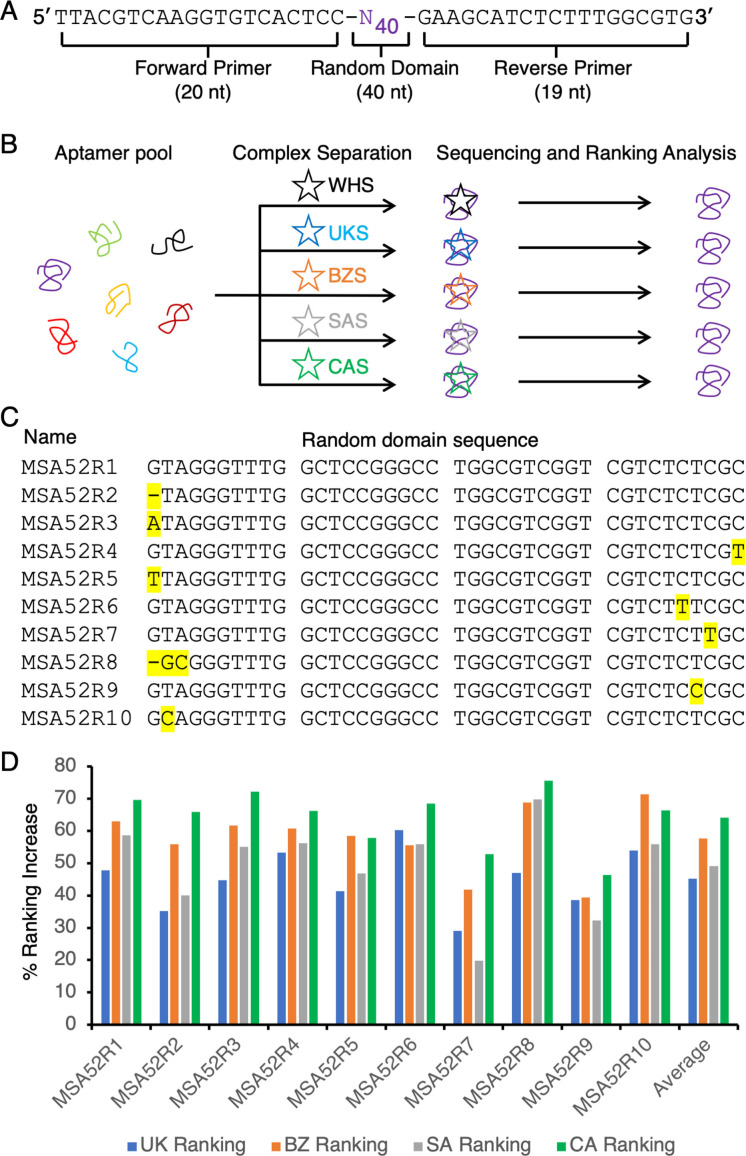
Discovery of MSA52. A) The sequence of the DNA library used for the original SELEX experiment. B) Schematic of reselection used for the discovery of MSA52. C) Top 10 members of the MSA52 aptamer family. Each point mutation in relation to the top ranked sequence is highlighted in yellow. D) Ranking increase of each of the top 10 sequences in the UK, BZ, SA and CA pools in comparison to their rankings in the WH pool. B.1.1.7 (blue), P.1 (red), B.1.351 (grey), and B.1.429 (green).

To test this idea and to quickly generate aptamer candidates for recognition of the spike proteins of diverse VoCs, we carried out five parallel one‐round SELEX experiments with the Generation‐13 pool and each of the five spike proteins that were commercially available at the time, including the original SARS‐CoV‐2 and its B.1.1.7, B.1.351, P.1 and B.1.429 variants (Figure 2B). Note that the spike protein for the B.1.617.2 Delta variant and B.1.1.529 Omicron variant were not yet available when this experiment was carried out. SELEX was done using electrophoretic mobility shift assays (EMSA) wherein each spike protein was first incubated with the DNA pool, followed by separation of the spike‐DNA complexes from the unbound DNA using native polyacrylamide gel electrophoresis. After elution from the gel, the bound DNA was amplified by PCR. The amplified DNA samples were then subjected to high‐throughput sequencing analysis as described in the Supporting Information.

We then compared the ranking of the top 100 aptamer sequences previously discovered (named MSA1 to MSA100) in each pool and found that the ranking of a particular aptamer, named MSA52, was significantly increased after reselection. MSA52 was ranked #52 in our original selection performed with the S1 subunit of WHS; however, the ranking increased to 46^th^, 24^th^, 19^th^, 17^th^, and 14^th^ in the pools established respectively with WHS, UKS, BZS, SAS and CAS, respectively, suggesting that MSA52 competed favorably with the top‐ranking sequences.

There are a total of 321 members of the MSA52 aptamer family; the sequences, ranking within each pool and frequency values of the top 100 members are provided in Table S2. Figure 2C lists the top 10 sequences in the family (which are named MSA52R1‐10). We calculated the ranking increase of each of the top 10 sequences in the UK, BZ, SA and CA pools in comparison to their rankings in the WH pool. Interestingly, all these top 10 sequences exhibit ∼50 % ranking increases in each variant pool (Figure 2D), once again confirming that the members of this aptamer family competed well for binding to all the four variants used for the SELEX experiment.

We also performed a t‐test to compare the ratio of frequencies of the top 15 members of the MSA52 cluster in each of the 5 selections vs. the initial (round 13) frequencies (Figure S2 and Table S3). These sequences were indeed significantly enriched for the pools selected with variant protein targets (P<0.0001). In contrast, the difference between the round 13 reference population and the WHS pool is insignificant (P=0.7718; the geometric mean of ratio and 95 % CI can be found in Table S3). Taken together, the sequencing data analysis pointed to the possibility that MSA52 may function as a universal spike protein binding aptamer that is insensitive to the mutations observed in spike proteins of the B.1.1.7, B.1.351, P.1 and B.1.429 variants.

We assessed the binding affinity of MSA52 (also known as MSA52R1 in Figure 2C) for the full trimeric spike (TS) proteins of SARS‐CoV‐2 and its B.1.1.7, B.1.351, P.1, B.1.429 variants using dot blot assays. Representative dot blots from these experiments are shown in Figure 3A, and binding curves of bound fraction vs. protein concentration are plotted in Figure 3B to derive the *K*_d_ values. As expected, MSA52 showed strong binding to all five TS proteins, the *K*_d_ values varied between only 3.6–10.2 nM for the five TS variants. The aptamer showed nearly identical affinity for WHS (3.6 nM), UKS (B.1.1.7S; 3.8 nM), and CAS (B.1.429S; 3.8 nM), and slightly reduced but still excellent affinity for SAS (B.1.351S; 8.5 nM) and BZS (P.1S; 10.2 nM). The binding data is consistent with the selection outcome, suggesting that the increase of the frequency of MSA52 and its related family members was likely a result of its high affinity for the spike proteins of the variants.

**Figure 3:**
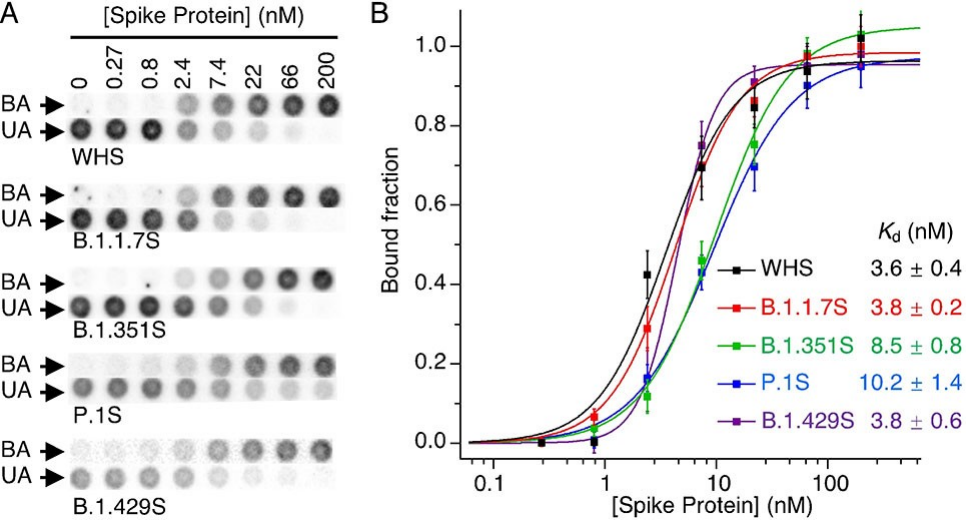
Assessment of the binding affinity of MSA52 for trimeric spike proteins of SARS‐CoV‐2 and its B.1.1.7, B.1.351, P.1, B.1.429 variants using dot blot assays. A) Representative dot blot results showing binding of MSA52 to the five trimeric spike proteins. B) Binding curves used to derive the *K*_d_ values for MSA52 for the trimeric spike protein.

As noted above, the spike proteins of the Indian VoCs were not available when we conducted the one‐round SELEX experiments. Since then, the spike proteins of both the Kappa (B.1.617.1) and Delta (B.1.617.2) variants as well as the Omicron (B.1.1.529) variant became available, giving us the opportunity to assess whether the MSA52 aptamer could detect emerging variants not known at the time of selection. Using dot blot assays (Figure 4A), we tested the binding of MSA52 to the TS proteins of these three new variants. Binding curves (Figure 4B) showed that the aptamer binds to B.1.617.1S, B.1.617.2S and B.1.1.529S with *K*_d_ values of 2.8 nM, 3.7 nM and 6.2 nM, respectively, which were very close to the *K*_d_ value observed for WHS (*K*_d_=3.6 nM). This observation further confirms MSA52 is a “universal” aptamer for spike protein recognition, as it can recognize all 7 spike protein variants of SARS‐CoV‐2 we have tested so far, including those not included during the one‐round selection.

**Figure 4:**
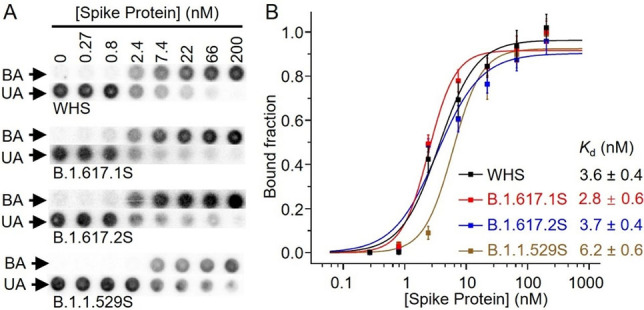
Assessment of binding affinity of MSA52 for TS proteins of the original SARS‐CoV‐2 virus and its B.1.617.1, B.1.617.2 and B.1.1.529 variants. A) Representative dot blot results showing the binding of MSA52 to the four TS proteins. B) Binding curves used to derive the *K*_d_ values for MSA52 for the TS proteins.

To better understand the differences in the MSA1, MSA5 and MSA52 binding properties, we conducted competition assays to examine whether the binding sites on the S1 protein of SARS‐CoV‐2 overlapped for all three aptamers. In this experiment, one aptamer (the aptamer that was being competed against) was radioactively labeled (labeled with * in Figure 5) and used at 2.5 nM, while the concentration of the other aptamer (the aptamer that was competing) was varied between 0–160 nM. Note that the S1 protein was used at 5 nM. Representative dot blot assays from these competitions are shown in Figure S3.

**Figure 5:**
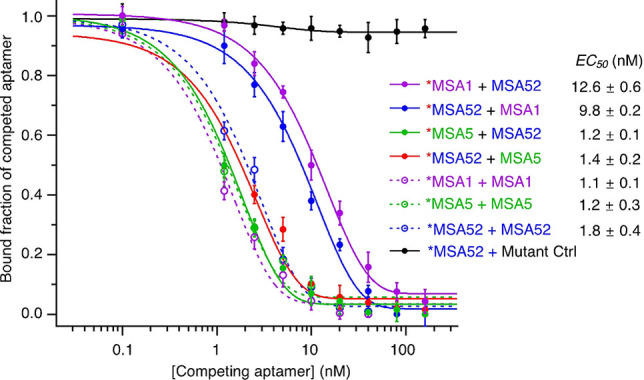
Competing assays to probe the binding site on the S1 protein of SARS‐CoV‐2 by of MSA52, MSA1 and MSA5.

Two controls were done to validate the competition: an inactive aptamer control (Mutant Ctrl, Figure 5; the sequences of all the DNA molecules used in this study are provided in Table S4) that should not compete with any aptamer; and self‐competition between radioactive and nonradioactive versions of the same aptamer (*MSA1+MSA1, *MSA5+MSA5, *MSA52+MSA52). The inactive aptamer was indeed unable to compete with MSA52, even at high concentrations (Figure 5; Figure S3). In contrast, each radioactive aptamer can be successfully competed out (Figure S3) by the non‐radioactive aptamer in each self‐competition, with *EC*_50_ values varying between 1–2 nM (Figure 5).

Based on these control experiments and the competitions between MSA1 and MSA52 or between MSA5 and MSA52, we can conclude that: (1) MSA5 competes very well with MSA52 (*EC*_50_=1.2 and 1.4, respectively for *MSA5+MSA52 and *MSA52+MSA5); (2) MSA1 and MSA52 do not compete well with each other (*EC*_50_=12.6 and 9.8, respectively for *MSA1+MSA52 and *MSA52+MSA1). Taking the data presented in Figure 1 into consideration, we interpret the competition results to infer that the binding sites of MSA52 and MSA1 do not significantly overlap while those of MSA52 and MSA5 show significant overlap, though it seems that MSA52 binds to amino acids of the S1 protein that have not been mutated in the current variants of concern that we tested.

We then examined the specificity of MSA52 by evaluating its binding to bovine serum albumin (BSA), human‐*α*‐thrombin, the RBD and spike (S) proteins of SARS‐CoV‐1, and the RBD proteins of MERS and three seasonal coronavirus 229E, NL63, OC43; the data are provided in Figure S4. MSA52 exhibits no binding to BSA and thrombin, SARS1‐RBD, MERS‐RBD or NL63‐RBD, but shows very weak binding to the spike protein of SARS‐CoV‐1, 229E‐RBD and OC43‐RBD (Figure S4). However, the binding affinity of MSA52 for these targets was very poor, with *K*_d_ values greater than 150 nM for the spike protein of SARS‐CoV‐1 and greater than 200 nM for 229E‐RBD and OC43‐RBD (Figure S5). Given that SARS‐CoV‐1 is no longer in circulation and that the aptamer does not efficiently recognize 229E and OC43, it is clear that MS52 shows sufficient selectivity for SARS‐CoV‐2.

Next, we tested MSA52 for binding to several pseudotyped SARS‐CoV‐2 lentiviruses (PVs) that were engineered to display the full trimeric S‐proteins of SARS‐CoV‐2 variants within the viral envelope. These pseudotyped viruses mimic SARS‐CoV‐2, but they cannot replicate themselves in human cells, allowing them to be handled in biosafety‐level‐2 labs. We performed the dot blot assays with eight different PVs containing the spike proteins of the original SARS‐CoV‐2 as well as the B.1.1.7, B.1.351, P.1, B.1.429, B.1.617.1, B.1.617.2, and B.1.617.2.1 (Delta Plus) variants (note the Omicron PV was not yet available) (Figure S6). The same lentivirus that lacks the S‐protein was used as a control virus (CV) for this experiment. MSA52 was found to recognize all these PVs with similar affinity but not the CV (Figure 6A). Excellent *K*_d_ values were observed for all eight PVs, which ranged from 18.4 pM (WH‐PV) to 49.0 pM (B.1.617.1‐PV). The increased affinity in comparison to the purified spike proteins can be explained by the fact that each viral particle carries many copies of the S‐protein. The copy number of the spike protein on the surface of the SARS‐CoV‐2 viruses has been reported to be ∼30;[^28^, ^29^] however, the copy number on the viral particles of the PVs we used in this experiment has not been reported. If we assume each PV virus carries 100 copies of the S‐protein, the protein‐equivalent *K*_d_ values are estimated to be in the range of 1.84 nM and 4.9 nM, which are close to the *K*_d_ values for the spike proteins (Figures 3 and 4). More Importantly, MSA52 can recognize fully functional spike proteins of the original SARS‐CoV‐2 as well as multiple VoCs, even though this aptamer was selected using the purified spike protein from the original virus.

**Figure 6:**
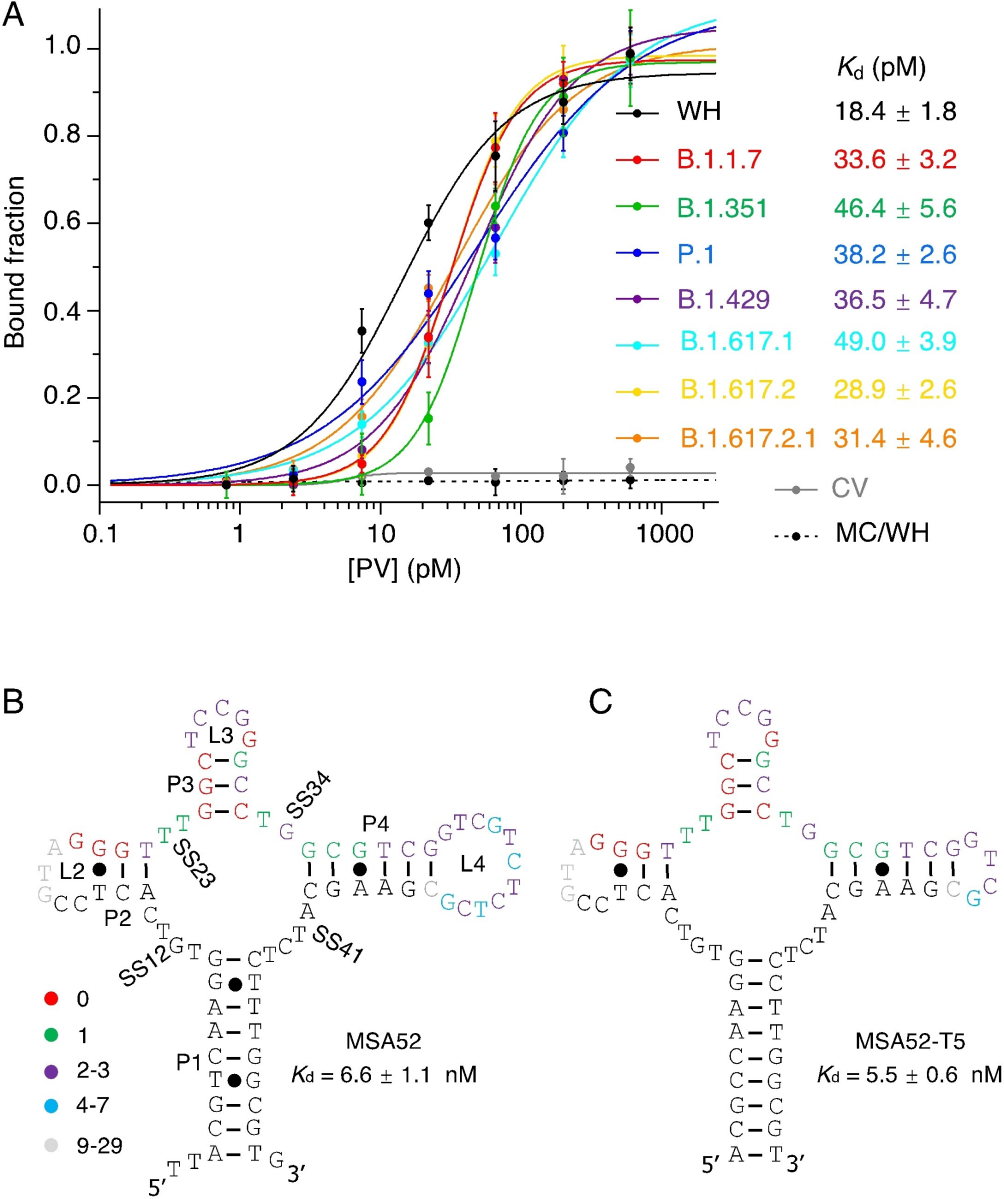
A) Assessment of binding affinity of MSA52 for lentiviruses pseudotyped to express the spike proteins of the original SARS‐CoV‐2 virus (WH) as well as B.1.1.7, B.1.351, P.1, B.1.429, B.1.617.1, B.1.617.2, and B.1.617.2.1 variants. CV: Control Lentivirus. MC: Mutant Control. Proposed secondary structures of B) full‐length MSA52 and C) a minimized version of MSA52 (named MSA52T5). *K*_d_ values represent the binding affinity for SARS‐CoV‐2 S1 protein.

The predicted structure of MSA52 is provided in Figure 6B. The overall structure contains 4 pairing elements (9‐bp P1, 3‐bp P2, 3‐bp P3 and 6‐bp P4) and 5 unpaired elements (4‐nt SS12, 2‐nt SS23, 2‐nt SS34, 4‐nt SS41, 6‐nt L2, 5‐nt L3, 11‐nt L4). Five truncation mutants (Figure S7) were examined by the dot blot assay (named MSA5‐T1 to MSA5‐T5, respectively) for the binding activity to the S1 protein of the original SARS‐CoV‐2 virus. The *K*_d_ value of the full‐length MSA52 was first determined to be 6.6 nM. Removing P2‐L2 (MSA52‐T1; loss of 10 nucleotides; *K*_d_ of 61.8 nM), P3‐L3 (MSA52‐T2; loss of 9 nucleotides; *K*_d_ of 53.4 nM), and P4‐L4 (MSA52‐T3; loss of 21 nucleotides; *K*_d_ of 94.8 nM) led to mutants whose binding activities were significantly reduced (Figure S7). However, L4 can be reduced to 4 nucleotides without activity reduction (MSA52‐T4; loss of 7 nucleotides; *K*_d_ of 6.0 nM). Finally, the three unpaired nucleotides next to P1 can be removed and the T⋅G and G⋅T Wobble pairs can be converted to C‐G and G‐C Watson‐Crick pairs without any loss of activity (MSA52‐T5, Figure 6C; loss of 3 nucleotides and change of 2 nucleotides; *K*_d_ of 5.5 nM). The results above support the proposed four‐way junction structure of MSA52.

Low‐level random mutations typically occur during the PCR step of the selection process. However, nucleotides whose bases play important roles in the structure and/or binding function of the aptamer show fewer mutations than structurally and/or functionally unimportant bases. We color‐coded the nucleotides in the original random‐sequence domain after performing sequence alignment of the top 100 members of the MSA52 family (Figure 6B): red‐absolutely conserved (0 mutations observed within the top 100 sequences); green ‐ highly conserved (1 mutation only); purple‐somewhat conserved (2–3 mutations); light blue‐less conserved (4–7 mutations); grey‐least conserved (9–29 mutations). The conservation pattern seen with the sequence of MSA52 is consistent with the truncation data: any truncation mutant that loses the red and green nucleotides experienced significantly reduced binding affinity. We further hypothesize that the nucleotides that directly engage the spike protein for molecular recognition are located within the 22‐nt segment starting with the red GGG residues within P2‐L2 and ending with the green GCG residues within P4. More detailed structural and functional analysis of these nucleotides constitutes a future research interest via the examination of more mutated sequence constructs.

We next conducted an enzyme‐linked aptamer binding assay (ELABA) to demonstrate the analytical utility of MSA52 for the detection of pseudotyped lentiviruses of the original SARS‐CoV‐2 (WH) and the B.1.1.7, B.1.351, P.1 and B.1.617.2 variants. MSA1 and three other reported aptamers were also tested for comparison with MSA52. Because each viral particle carries multiple spike proteins on its surface, we designed a sandwich assay that uses two identical biotinylated aptamers to bind a single viral particle (Figure 7A). Each aptamer was biotinylated at the 5′ end and immobilized onto a 96‐well microtiter plate coated with streptavidin. The second biotinylated aptamer was tagged with horseradish peroxidase (HRP) conjugated to streptavidin. The presence of viral particles led to immobilization of HRP onto the plate, which oxidized 3,3′,5,5′‐tetramethylbenzidine (TMB) in the presence of H_2_O_2_ (Figure 7A). After quenching the reaction with H_2_SO_4_, the blue‐colored oxidized TMB turned yellow and was measured at 450 nm. BSA protein was used as the blank control to subtract background for each aptamer and a polyA_25_ sequence was tested as a DNA control. Figure 7B plots the absorbance at 450 nm (A_450_) in binding reaction mixtures containing a specific aptamer and pseudotyped lentivirus (PL, 2 pM). MSA52 was able to produce high signals (A_450_ greater than 0.05) for all five variants; in contrast, high signals were only observed with MSA1 for two variants (B.1.1.7 and B.1.617.2), SARS2‐AR10 for two variants (WH and B.1.1.7), S1P for one variant (B.1.1.7). nCoV‐S1‐A1 produced weak signals for all five variants. These observations are also consistent with the *K*_d_ values determined by dot blots (Table S1), which show that MSA52 is the only aptamer exhibiting consistently excellent *K*_d_ values for all the variants tested in the colorimetric assay.

**Figure 7:**
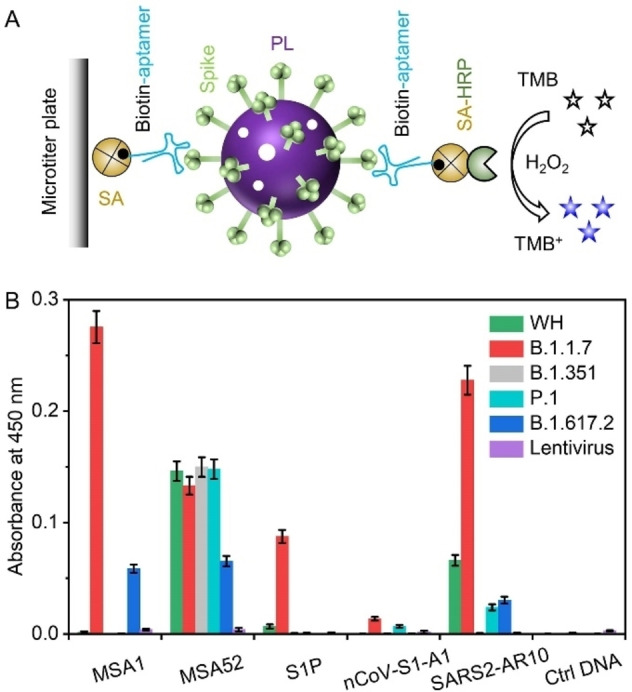
Sandwich assay of 5 aptamers for the detection of pseudotyped lentiviruses (PL) expressing the original SARS‐CoV‐2 (WH) and the B.1.1.7, B.1.351, P.1 and B.1.617.2 variants. A) Schematic illustration of the working principle. B) Absorbance at 450 nm produced by HRP tagged aptamers in oxidation of TMB in the presence of H_2_O_2_, followed by quenching with H_2_SO_4_. SA: streptavidin. HRP: horseradish peroxidase.

In summary, we have performed parallel one‐round selection using an aptamer pool from a previous SELEX experiment with the wild‐type S1 protein of SARS‐CoV‐2, followed by high‐throughput DNA sequencing and bioinformatic analysis, in search for DNA aptamers that universally (i. e., non‐discriminatively) recognize spike proteins of SARS‐CoV‐2 variants. This effort has led to the discovery of a unique DNA aptamer, named MSA52, that can bind spike proteins of the wildtype SARS‐CoV‐2 and its seven current variants of concern, including B.1.1.7, B.1.351, P.1, B.1.429, B.1.617.1, B.1.617.2 and B.1.1.529. This aptamer was discovered using reselection against four variants of the SARS‐CoV‐2 spike protein, but was observed to show high affinity binding to both these four variants as well as three variants not present during reselection, indicating universally high affinity for both known and emerging VoCs. The entire discovery process (SELEX, sequencing and bioinformatic analysis) was very rapid, taking less than a week. Competition assays suggested that this unique aptamer likely bound to key amino acids present in all variants, allowing universal recognition of the spike proteins of the current and emerging variants of concern, making it well‐suited as a molecular recognition element for developing diagnostic and therapeutic solutions to this constantly evolving coronavirus.

## Conflict of interest

The authors declare no conflict of interest.

## Supporting information

As a service to our authors and readers, this journal provides supporting information supplied by the authors. Such materials are peer reviewed and may be re‐organized for online delivery, but are not copy‐edited or typeset. Technical support issues arising from supporting information (other than missing files) should be addressed to the authors.

Supporting Information

## Data Availability

The data that support the findings of this study are available in the supplementary material of this article.
