# Supplementary material for: A Universal DNA Aptamer that Recognizes Spike Proteins of Diverse SARS‐CoV‐2 Variants of Concern
Source: Chemistry. 2022 Feb 18;28(15):e202200078. doi: 10.1002/chem.202200078 (PMC9015322; doi:10.1002/chem.202200078)
Supplement: Supplementary file 1 — Supporting Information [file CHEM-28-0-s001.pdf]

Chemistry–A European Journal

Supporting Information

A Universal DNA Aptamer that Recognizes Spike Proteins of Diverse SARS-CoV-2 Variants of Concern

Zijie Zhang, Jiuxing Li, Jimmy Gu, Ryan Amini, Hannah D. Stacey, Jann C. Ang, Dawn White, Carlos D. M. Filipe, Karen Mossman, Matthew S. Miller, Bruno J. Salena, Deborah Yamamura, Payel Sen, Leyla Soleymani, John D. Brennan,* and Yingfu Li*

SECTION A: MATERIALS AND METHODS

A1. Materials and Reagents

DNA oligonucleotides were obtained from Integrated DNA Technologies (IDT) and purified by standard 10% denaturing (8 M urea) polyacrylamide gel electrophoresis (dPAGE) before use. The sequences are listed in Table S2 and S4. The Wuhan SARS-CoV-2 spike protein subunit S1 (catalog number: 40591-V08B1) was purchased from Sino Biological Inc. The Wuhan SARS-CoV-2 full spike protein (WHS, molecular weight 140 kDa), spike-pseudotyped lentiviruses for Wuhan SARS-CoV-2 (WH), variant B.1.351, variant P.1 and control lentivirus were prepared using standard methods (see details below). The full spike proteins for the variants B.1.1.7 (catalog number: SPN-C52H6), B.1.617.1 (SPN-C52Hr), B.1.617.2 (SPN-C52He) and B.1.1.529 (SPN-C52Hz) were expressed from human 293 cells (HEK293) and obtained from Acro Biosystems. The spike proteins for the variants B.1.351 (510333-1), P.1 (100989-1) and B.1.429 (101057) were expressed from human 293 cells (HEK293) and obtained from BPS Biosciences Inc. The spike and RBD proteins for SARS-CoV-1, MERS and seasonal coronavirus 229E, NL63 and OC43 were provided by Dr. Miller's lab at McMaster University. The concentrations of the proteins were quantified using bicinchoninic acid (BCA) protein assay kits from Thermo Scientific (Catalog number: 23225). The SARS-CoV-2 spike-pseudotyped lentivirus for the variant B1.1.7 (catalog number: 78112-1), B.1.429 (78172-1), B.1.617.1 (78205-1), B.1.617.2 (78216-1) and B.1.617.2.1 (78219-1) were obtained from BPS Bioscience. Nitrocellulose blotting membranes (catalog No. 10600125) were purchased from GE Healthcare Inc. Nylon hybridization transfer membranes (NEF994001PK) were purchased from PerkinElmer Inc. (Woodbridge, ON, Canada). T4 polynucleotide kinase (PNK), adenosine triphosphate (ATP) and deoxyribonucleoside 5'-triphosphates (dNTPs) were purchased from Thermo Scientific (Ottawa, ON, Canada). γ-[^{32}P]-ATP was acquired from PerkinElmer. Bovine serum albumin (BSA) and human thrombin were purchased from Sigma-Aldrich (Oakville, Canada). 4-(2-hydroxyethyl)-1-piperazineethanesulfonic acid (HEPES), sodium chloride, magnesium chloride, Tween-20 and all other chemicals were purchased from Sigma-Aldrich (Oakville, Canada) and used without further purification. Milli-Q water was used for all the experiments.

A2. Radiolabelling of DNA Aptamers

DNA aptamers were labeled with γ-[^{32}P] ATP at the 5′-end using PNK reactions according to the manufacturer's protocol. Briefly, 2 μL of 1 μM DNA aptamers were mixed with 2 μL of γ-[^{32}P] ATP, 1 μL of 10 × PNK reaction buffer A, 10 U (U: unit) of PNK and 4 μL water. The mixture was incubated at 37 °C for 20 min, and then purified by 10% dPAGE.

A3. Preparation of Recombinant Full Trimeric Spike Protein

A detailed protocol outlining protein production can be found in Stadlbauer et al. 2020.[1] The plasmid encoding the mammalian cell codon optimized sequence for Wuhan SARS-CoV-2 full length spike protein was generously gifted from the lab of Dr. Florian Krammer (Ichan School of Medicine, NYC).[2] In brief, proteins were produced in Expi293 cells (ThermoFisher Scientific) using the manufacturers' instructions. When culture viability reached 40%, supernatants were collected and spun at 500 g for 5 minutes. The supernatant was then incubated with 1 ml

of Ni-NTA agarose (Qiagen) per 25 ml of transfected cell supernatant overnight, with shaking, at 4 °C. The following day 10 ml polypropylene gravity flow columns (Qiagen) were used to elute the protein. Spike proteins were concentrated in 50 kDa Amicon centrifugal units (Millipore) prior to being resuspended in phosphate buffered saline (PBS).

A4. Preparation of Lentivirus

Wuhan SARS-CoV-2 S protein pseudotyped lentivirus was produced as described by Crawford et al.[3] SARS-Related Coronavirus 2, Wuhan-Hu-1 Spike-Pseudotyped Lentiviral Kit (BEI catalog number NR-52948) was obtained through BEI resources, National Institute of Allergy and Infectious Diseases, National Institutes of Health. In brief, HEK293T cells were seeded in 15 cm dishes at 1.1 x 10^7 cells/mL in 15 mL of standard Dulbecco's Modified Eagle Medium (DMEM). 16 - 24 hours post seeding, cells were co-transfected with HDM-nCoV-Spike-IDTopt-ALAYT (BEI catalog number NR-52515), pHAGE-CMV-Luc2-IRES-ZsGreen-W (BEI catalog number NR-52516), HDM-Hgpm2 (BEI catalog number NR-52517), HDM-tat1b (BEI catalog number NR-52518) and pRC-CMV-Rev1b (BEI catalog NR-52519). 18 - 24 h post-transfection the media was replaced with full DMEM and 60 h post transfection, the supernatant was collected and filtered with a 0.45 μm filter and stored at -80 °C until future use. For purification, 40 mL of supernatant was concentrated by spinning at 19,400 rpm for 2 h. The resulting pellet was resuspended in 400 μl of HBSS, followed by 15 min of continuous vortexing at room temperature. Protein concentration was confirmed by the BCA assay.

A5. Rapid Selection of Aptamers for Spike Proteins of SARS-CoV-2 Variants

The one-round selection processes were carried out by native gel-based methods using a pre-enriched DNA pool for the spike protein of wildtype SARS-CoV-2 after 13 rounds of selection.[4] Briefly, the FAM-labelled pre-enriched DNA pool was diluted in 10 µL selection buffer (1× SB; 50 mM HEPES, 6 mM KCl, 150 mM NaCl, 2.5 mM $CaCl_2$, 2.5 mM $MgCl_2$, 0.01% v/v Tween-20, pH 7.4) to 100 nM, followed by heating at 90 °C for 5 min and annealing at room temperature for 10 min. Spike proteins (10 µL, 800 nM, in 1× SB) of different SARS-CoV-2 variants were then mixed with the DNA pool and incubated at 23 °C for 30 min. The DNA bound with spike proteins was separated from unbound DNA using 10% (v/v) native PAGE, which was imaged using a Typhoon imaging system (Typhoon™ FLA 9500, GE Healthcare, USA). Afterward, the bound DNA was cut and eluted from the gel by incubating in 1× Taq buffer (200 µL, 50 mM KCl, 10 mM Tris-HCl, 1.5 mM $MgCl_2$, 1% v/v Triton X-100, pH 9.0) at 23 °C for 20 min. The DNA in the supernatant was amplified by PCR, after the addition of FP1 (10 µL, 10 µM), RP1 (10 µL, 10 µM), Taq DNA polymerase (2 µL, 5 U/µL), and dNTPs (20 µL, 2 mM). The PCR temperature profile was set as follows: preheating at 94 °C for 30 s; temperature cycles of 94 °C for 30 s, 50 °C for 30 s, and 72 °C for 30 s; annealing at 72 °C for 5 min. The PCR products were then further amplified by PCR using sequencing primers and then analyzed using the MiSeq (Illumina) sequencing platform. The selection processes were simultaneously carried out for WHS, UKS, SAS, BZS and CAS.

A6. Sequencing Data Analysis

Sequencing samples were prepared from each parallel SELEX experiment by PCR tagging with Illumina sequencing primers. Samples were size purified by agarose gel electrophoresis prior to being quantified by measuring absorbance at 260 nm. Tagged samples were pooled and paired-end sequenced on an Illumina MiSeq high-throughput DNA sequencer. Sequence data processing was performed on a Windows 10 computer running Ubuntu 20.04 under WSL2. Raw paired-end reads were trimmed of sequencing and library primers using cutadapt 3.4.[5] Trimmed paired-end reads were then: 1) merged into a consensus sense read; 2) dereplicated; and, 3) clustered at 90% identity using USEARCH v11.0.667_i86linux32.[6] Sequence frequencies and ranking lists were generated using custom Python scripts. Multiple sequence alignments were performed using MUSCLE v3.8.1551 and converted to sequence logos using WebLogo 3.7.8.[7,8] Processed sequencing data and cluster linkage data were stored on a MySQL 8.0.22 database. Analysis of sequence copy number, frequency, cluster linkage and data plots were performed using the database and Microsoft Excel.

DNA pools selected against variant Spike proteins were each tagged with a unique barcode and paired-end sequenced on an Illumina MiSeq. Pools were trimmed of primers using Cutadapt 3.4.[5] Trimmed reads were paired-end merged using USEARCH v11.0.667_i86linux32,[6] only sequences with perfect complementarity were returned for further analysis. Trimmed and merged reads were then dereplicated and annotated with a copy number using USEARCH to yield a unique, copy number ranked list of random domain sequences for each pool. To ensure consistency of clustering when comparing across pools, dereplicated sequences from each pool were combined into a descending ranked master list upon which clustering was performed using USEARCH (cluster_smallmem with arguments -id 0.9, -query_cov 0.9, -target_cov 0.9) to yield a list of reference sequences representing clusters of sequences which share 90% sequence identity with the reference sequence. Table S2 lists all mutant sequences sharing MSA52 as the reference sequence (the "MSA52 Cluster") with their ranks and frequencies in the variant Spike selection pools, if detected.

A7. Dot Blot Binding Assays with Spike Protein

Dot blot assays were performed by using a Whatman Minifold-1 96-well apparatus and a vacuum pump. Before experiments, nitrocellulose membranes and nylon membranes were incubated in 1× SB buffer for 1 h. γ-[^{32}P] labelled DNA aptamers (1 nM) were dissolved in the binding buffer and heated at 90 °C for 5 min, and then cooled at room temperature for 20 min. Spike proteins were dissolved and diluted in the same buffer. 5 μL of the above aptamer solution was mixed with 15 μL of spike protein with different concentrations. The mixture was incubated at room temperature for 1 h. The dot blot apparatus was assembled with a nitrocellulose membrane on the top, a nylon membrane in the middle and a wetted Whatman paper in the bottom. After washing each well with 100 μL of binding buffer, the binding mixtures were loaded and drained by the vacuum pump (force: 550 mmHg for 8 seconds). The wells were then washed twice with 100 μL binding buffer. The membranes were imaged using a Typhoon 9200 imager (GE Healthcare) and analyzed using Image J software.[9] Each binding assay was performed 3 times. The bound fraction was quantified and plotted against the concentration of the protein. The K_d

values were derived via curve fitting using Origin 8.0 using the equation $Y = B_{max}X/(K_d + X)$ (Y is the bound fraction of aptamer with protein, B_{max} is the maximum bound fraction of aptamer, and X is protein concentration).

A8. Dot Blot Binding Assays with SARS-CoV-2 Spike-Pseudotyped Lentivirus

Dot blot assays with SARS-CoV-2 spike-pseudotyped lentivirus and the control lentivirus without spike protein were performed using the same procedure as described above except: the aptamer solutions were incubated with different concentrations of viruses (0 - 600 pM of viral particles; corresponding to $0 – 3 \times 10^{11}$ cp mL^{-1}) for 10 min, followed by performing dot blot assays.

A9. ELABA for pseudotyped lentivirus

The ELABA (enzyme-linked aptamer binding assay) for different pseudotyped lentivirus was conducted on a Pierce™ streptavidin-coated microtiter plate (Thermo Scientific™, catalog No. 15121). The plate wells were washed three times with 200 µL 1× SB buffer (50 mM HEPES, 6 mM KCl, 150 mM NaCl, 2.5 mM $CaCl_2$, 2.5 mM $MgCl_2$, 0.01% v/v Tween-20, pH 7.4) after the binding of each reagent. First, the plate wells were blocked with blocking buffer (300 µL, 1× SB buffer, 10% w/v BSA) by incubation at 37 °C for 1 h. Biotinylated aptamers (100 µL, 200 nM) in dilution buffer (1× SB with 0.1% w/v BSA) were then added to the wells and incubated at 22 °C for 30 min. The pseudotyped lentivirus (100 µL, 2 pM) in dilution buffer was then introduced and captured by aptamers on the plate, followed by incubation at 22 °C for 1 h. Next, the same biotinylated aptamer (100 µL, 200 nM) was added again and used as a reporter aptamer to bind with the virus with incubation at 22 °C for 30 min. Subsequently, streptavidin-HRP (100 µL, 1:2000 dilution, New England Biolabs, catalog No. 3999S) in dilution buffer was introduced to bind to the reporter aptamer with incubation at 22 °C for 30 min. Finally, 1-Step™ Ultra TMB-ELISA Substrate Solution (100 µL, Thermo Scientific™, catalog No. 34028) was introduced and reacted at 22 °C for 20 min. H_2SO_4 (20 µL, 2 M) was used to terminate the catalytic reaction, converting TMB to its oxidized product, which was measured by a plate reader (Tecan, Switzerland) at 450 nm.

SECTION B: SUPPORTING TABLES

Table S1. Affinity (*K_d*) summary of eight reported aptamers binding to WHS and variant trimeric spike proteins.

**Aptamer name**	***K_d* (nM)**				References
	**WHS**	**B.1.1.7S**	**B.1.351S**	**P.1S**	
**MSA52**	**3.6 ± 0.4**	**3.8 ± 0.2**	**8.5 ± 0.8**	**10.2 ± 1.4**	**This work**
MSA1	19.8 ± 2.6	1.2 ± 0.3	> 200	75.2 ± 6.1	[4]
MSA5	5.6 ± 0.6	4.2 ± 0.4	8.2 ± 0.8	52.3 ± 8.2	[4]
CoV2-RBD-1	37.4 ± 3.5	8.1 ± 0.4	78.9 ± 8.2	78.6 ± 7.4	[10]
CoV2-2	26.6 ± 3.1	15.2 ± 1.6	68.8 ± 7.7	78.1± 9.2	[11]
Aptamer-1	4.7 ± 0.9	8.5 ± 1.2	51.2 ± 6.6	30.2 ± 2.4	[12]
S1P	18.2 ± 2.5	5.6 ± 0.3	62.6 ± 7.8	65.8 ± 5.8	[13]
nCoV-S1-A1	34.2 ± 5.0	28.4 ± 2.2	65.2 ± 5.8	43.5 ± 2.7	[14]
SARS2-AR10	8.6 ± 1.3	6.4 ± 0.5	69.2 ± 7.9	42.4 ± 4.5	[15]

Table S2. DNA sequences in the combined pools selected for WHS, UKS, BZS, SAS, and CAS ranked by their frequency.

Sequence[a]	Rank[b]					Frequency (10^{-6})				
	WHS	UKS	BZS	SAS	CAS	WHS	UKS	BZS	SAS	CAS
GTAGGGTTTGGCTCCGGGCCTGGCGTCGGTCGTCTCTCGC	46	24	17	19	14	1382	3203	4447	4294	3860
-TAGGGTTTGGCTCCGGGCCTGGCGTCGGTCGTCTCTCGC	455	295	201	273	155	95	171	280	206	293
ATAGGGTTTGGCTCCGGGCCTGGCGTCGGTCGTCTCTCGC	460	254	176	207	128	94	196	323	293	336
GTAGGGTTTGGCTCCGGGCCTGGCGTCGGTCGTCTCTCGT	757	354	297	332	256	54	139	196	161	164
TTAGGGTTTGGCTCCGGGCCTGGCGTCGGTCGTCTCTCGC	1128	662	468	599	475	34	60	109	83	74
GTAGGGTTTGGCTCCGGGCCTGGCGTCGGTCGTCTTTCGC	1756	697	780	775	553	17	55	59	61	57
GTAGGGTTTGGCTCCGGGCCTGGCGTCGGTCGTCTCTTGC	1851	1313	1077	1484	874	16	23	39	25	29
G-CGGGTTTGGCTCCGGGCCTGGCGTCGGTCGTCTCTCGC	2021	1070	631	612	492	13	31	77	81	69
GTAGGGTTTGGCTCCGGGCCTGGCGTCGGTCGTCTCCCGC	2041	1253	1238	1383	1095	13	24	32	28	21
GCAGGGTTTGGCTCCGGGCCTGGCGTCGGTCGTCTCTCGC	2172	1000	620	957	729	12	34	79	46	38
CTAGGGTTTGGCTCCGGGCCTGGCGTCGGTCGTCTCTCGC	2203	2663	1296	1595	961	12	9	31	23	24
GTAGGGTTTGGCTCCGGGCCTGGCGTCGGTTGTCTCTCGC	2785	1168	1700	1800	1331	8	27	22	19	16
GTAGGGTTTGGCTCCGGGCCTGGCGTCGGTCATCTCTCGC	2788	1471	1797	1602	983	8	20	20	23	24
GTAGGGTTTGGCTCCGGGCCTGGCGTCGGTCGTCCCTCGC	2790	1252	1200	1483	1040	8	24	34	25	22
GTGGGGTTTGGCTCCGGGCCTGGCGTCGGTCGTCTCTCGC	3110	2141	1917	2129	1042	7	12	18	16	22
GTAGGGTTTGGCTCCGGGCCTGGCGTAGGTCGTCTCTCGC	3132	2134	1698	2818	1228	7	12	22	11	17
GAAGGGTTTGGCTCCGGGCCTGGCGTCGGTCGTCTCTCGC	3140	1763	2434	1791	1150	7	15	13	19	19
GTAGGGTTTGGCTCCGGGCCTGGCGTCGGTCGCCTCTCGC	3456	1663	1699	1728	2087	6	17	22	20	9
GTAGGGTTTGGCTCCGGGCCTGGCGTCGGTCGTCTCTCGA	3540	2135	2044	4571	1230	6	12	16	6	17
AC-GGGTTTGGCTCCGGGCCTGGCGTCGGTCGTCTCTCGC	4025	1238	1406	1518	854	5	24	27	24	29
GTAGGGTTTGGCTCCGGGCCTGGCGTCGGTCGTCTCTCTC	4054	1472	1201	2000	637	5	20	34	17	47
GTAGGGTTTGGCTCCGGGCCTGGCGTCGGTCGTTTCTCGC	4087	3157	3240	3431	1618	5	8	9	8	12
ATAGGGTTTGGCTCCGGGCCTGGCGTCGGTCGTCTCTCGT	4096	20213	7714	5234	1972	5	<1	4	5	9
-T-GGGTTTGGCTCCGGGCCTGGCGTCGGTCGTCTCTCGC	4101	2464	1731	4761	1182	5	11	22	6	19
GTAGGGTTTGGCTCAGGGCCTGGCGTCGGTCGTCTCTCGC	4844	8748	2042	26086	2082	5	3	16	<1	9
GTAGGGTTTGGCTCCGGGCCTGGCGTCGGTCGTCTC-CTC	4917	3156	2786	2427	1616	4	8	11	13	12
GTAGGGTTTGGCTCCGGGCCTGGCGTCGGTCCTCTCTCGC	5000	2692	1598	4569	1039	4	9	23	6	22
GTAGGGTTTGGCTCCGGGCCTGGCGACGGTCGTCTCTCGC	5026	2133	2225	5576	2083	4	12	14	5	9
GTAGGGTTTGGCTCCGGGCCTGGCGTCGGTCTTCTCTCGC	5102	5173	2463	3432	1041	4	5	13	8	22
-CAGGGTTTGGCTCCGGGCCTGGCGTCGGTCGTCTCTCGC	5144	21204	3766	24575	4702	4	<1	7	<1	3
--AGGGTTTGGCTCCGGGCCTGGCGTCGGTCGTCTCTCGC	6321	N	17877	N	N	4	N	<1	N	N
GTAGGGTTTGGCTCCGGACCTGGCGTCGGTCGTCTCTCGC	6417	22888	N	7532	11522	4	<1	N	4	<1
GTAGGGTTTGGCTCCGGGCCT-GCGTCGGTCGTCTCTCGC	6418	N	N	N	N	4	N	N	N	N
GTAGGGTTTGGCTCCGGGCCTGGCGTCGGTCGTCTCTCAC	6548	1664	1599	1999	1229	2	17	23	17	17
GTAGGGTTTGGCTCCGGGCCTGGCGTCGGTCGTCTCTCGG	6569	1777	3239	5580	2526	2	15	9	5	7
ACTGGGTTTGGCTCCGGGCCTGGCGTCGGTCGTCTCTCGC	6616	2559	1647	3268	9562	2	9	22	8	<1
GTAGGGTTTGGCTCCGGGCCTGGCGTCGGTCGTCTCTAGC	6617	3867	3238	5579	1096	2	6	9	5	21
GTAGGGCTTGGCTCCGGGCCTGGCGTCGGTCGTCTCTCGC	6623	8745	3235	2426	3269	2	3	9	13	5
GTAGGGTTTGGCTCCGGGCCTGGCGTCGGTCGTCTCTCCC	6671	2693	5228	4570	1617	2	9	5	6	12
ATGGGGTTTGGCTCCGGGCCTGGCGTCGGTCGTCTCTCGC	6737	8069	18301	3704	1988	2	3	<1	7	9
GTAGGGTTTGGCTCCGGGCCTGGCGTCGTTCGTCTCTCGC	6764	2136	2045	3433	834	2	12	16	8	31
GTAGGGTTTGGCTCCGGGCCTAGCGTCGGTCGTCTCTCGC	6794	22892	8593	26089	N	2	<1	4	<1	N
GTAGGGTTTGGCTCCGGGCCTGGCGTCGGTAGTCTCTCGC	6823	3865	3944	12354	2086	2	6	7	2	9
GGAGGGTTTGGCATCGGGCCTGGCGTCGGTCGTCTCTCGC	6825	N	20464	7463	3224	2	N	<1	4	5
GTAGGGTTTGGCTCCGGGCCTGGCTTCGGTCGTCTCTCGC	6826	2695	3949	26100	2091	2	9	7	<1	9
GTAGGGTTTGGCTCCGGGCCTGGCGCCGGTCGTCTCTCGC	6832	22896	8594	4568	2084	2	<1	4	6	9
GTAGGGTTTGGCTCCGGGCCCGGCGTCGGTCGTCTCTCGC	6843	22891	20717	12352	5032	2	<1	<1	2	3
-TGGGGTTTGGCTCCGGGCCTGGCGTCGGTCGTCTCTCGC	6861	24824	5568	7994	5381	2	<1	5	4	3
ACAGGGTTTGGCTCCGGGCCTGGCGTCGGTCGTCTCTCGC	7049	7672	3542	4195	1689	2	3	7	6	10
GTAGGGTCTGGCTCCGGGCCTGGCGTCGGTCGTCTCTCGC	8000	22882	8591	5574	11515	2	<1	4	5	<1
GTAGGGTTTGGCTCCGGGCCTGGCGTCGATCGTCTCTCGC	8110	22898	20721	26091	2525	2	<1	<1	<1	7
GTAGGGTTTGGCTCCGGGCCTGGCGTCGGTCGTCTCCCGT	8111	N	N	N	N	2	N	N	N	N
ATAGGGTTTGGCTCCGGGCCTGGCGTCGGTCGTTTCTCGC	9039	N	N	N	N	2	N	N	N	N
ATAGGGTTTGGCTCCGGGCCTGGCGTCGGTCTTCTCTCGC	9040	N	18072	N	4556	2	N	<1	N	3
GTAGGGTTTGGCTACGGGCCTGGCGTCGGTCGTCTCTCGC	9627	5170	2462	26085	3270	2	5	13	<1	5
GTAGGGTTTGGCTCCGGGCCTGGCGTCGGTCGTGTCTCGC	9628	22904	20727	N	11529	2	<1	<1	N	<1
-TAGGGTTTGGCTCCGGGCCTGGCGTCGTTCGTCTCTCGC	9730	N	21117	N	11857	2	N	<1	N	<1
-TAGGGTTTGGCTCCGGGCCTGGCGTCGGTCGTCTCTCGT	10472	5236	5295	2839	988	<1	5	5	11	24
TCGGGGGGGTTTGGCTCCGGGCCTGGCGTCGGTCGTCTCTCGC	10474	23821	4036	3124	1245	<1	<1	7	10	17
-TAGGGTTTGGCTCCGGGCCTGGCGTCGGTCGTCTTTCGC	10476	23260	8716	4607	2558	<1	<1	4	6	7
GTAGGGTTTGGCTCCGGGCCTGGCGTCGGCCGTCTCTCGC	10477	1927	2785	2591	1461	<1	14	11	12	14
GTTGGGTTTGGCTCCGGGCCTGGCGTCGGTCGTCTCTCGC	10491	8790	3250	2598	5060	<1	3	9	12	3
TCCGTAGGGTTTGGCTCCGGGCCTGGCGTCGGTCGTCTCTCGC	10536	5305	8812	7716	12173	<1	5	4	4	<1

GTAGGGTTTGGCCCCGGGCCTGGCGTCGGTCGTCTCTCGC	10556	8747	3237	3091	5029	<1	3	9	10	3
GTAGGGTTTGGCTCCAGGCCTGGCGTCGGTCGTCTCTCGC	10573	22887	20715	26087	5031	<1	<1	<1	<1	3
GTAGGGTTCGGCTCCGGGCCTGGCGTCGGTCGTCTCTCGC	10599	22883	2223	3090	5027	<1	<1	14	10	3
GTAGGGTTTGGCTCCGGGCCTGGCGTCGGTCGTCTCTC--	10665	2385	1915	3857	934	<1	11	18	7	26
-TCGGGTTTGGCTCCGGGCCTGGCGTCGGTCGTCTCTCGC	10669	N	8943	5744	12433	<1	N	4	5	<1
GTAGGGTTTGGCTCCGGGCCTGGCGTCGGACGTCTCTCGC	10678	3155	3943	2819	5034	<1	8	7	11	3
GTAGGGTTTGGCTCCGGGCCTGGCGTTGGTCGTCTCTCGC	10729	2694	2046	2428	2090	<1	9	16	13	9
GTAGGGTTTGGCTCCGGGCCTGGCGTCGGTCGTCTATCGC	10785	3866	8595	5578	1163	<1	6	4	5	19
ATAGGGTTTGGCTCCGGGCCTGGCGTCGGTTGTCTCTCGC	10794	20214	7717	6917	9824	<1	<1	4	4	<1
G-CGGGTTTGGCTCCGGGCCTGGCGTCGGTCGTCTCTCGT	10801	22512	3200	25741	4919	<1	<1	9	<1	3
GTAGGGTTTGGCTCCGGGCCTGGCGTCAGTCGTCTCTCGC	10895	22897	5225	5577	3272	<1	<1	5	5	5
GTAGGGTTTGGCTTCGGGCCTGGCGTCGGTCGTCTCTCGC	10897	22910	3243	7536	11532	<1	<1	9	4	<1
GTAGGGTTTGGCTCCGGGCCTGGCGTCGGTCGTCGCTCGC	10915	5172	20723	7534	N	<1	5	<1	4	N
GTAGGGTTTGGCTCCGGGCCTGGCGTCGGTCGACTCTCGC	10949	5171	20722	7533	11525	<1	5	<1	4	<1
GTAGGGTTTGGCTCCGGGCCTGGCGTCGGTCGTCACTCGC	10950	8751	5226	3430	2088	<1	3	5	8	9
GTAGGGTTTGGCTCCGGGCCTGGCGTCTGTCGTCTCTCGC	10951	3158	2787	3092	1164	<1	8	11	10	19
ATCGGGTTTGGCTCCGGGCCTGGCGTCGGTCGTCTCTCGC	10998	20292	4820	23795	9899	<1	<1	5	<1	<1
GTAGGGTTTGGCTCCGGGCCTGGCGTCGGTCGTCTC-CCC	11026	N	8596	12356	11527	<1	N	4	2	<1
-TAGGGTTTGGCTCCGGGCCTGGCGTCGGTCGTCTCTTGC	11030	3921	21116	26470	11856	<1	6	<1	<1	<1
TTAGGGTTTGGCTCCGGGCCTGGCGTCGGTCGTCTCTCGT	11041	26005	9590	13599	3557	<1	<1	4	2	5
GTAGGGTTTGGCTCTGGGCCTGGCGTCGGTCGTCTCTCGC	11050	8757	3242	3859	3275	<1	3	9	7	5
G—-GGGTTTGGCTCCGGGCCTGGCGTCGGTCGTCTCTCGC	11119	22782	8545	4552	11431	<1	<1	4	6	<1
AT-GGGTTTGGCTCCGGGCCTGGCGTCGGTCGTCTCTCGC	11158	20431	18316	11470	9984	<1	<1	<1	2	<1
GTAGGGTTTGGCTCCGGGCCTGACGTCGGTCGTCTCTCGC	11172	N	20718	12353	11523	<1	N	<1	2	<1
-T-GGGATTGGCTCCGGGCCTGGCGTCGGTCGTCTCTCGC	11177	24782	N	7980	13081	<1	<1	N	4	<1
GTAGGGTTTGGCTCCGGGTCTGGCGTCGGTCGTCTCTCGC	11192	2137	8597	5585	11531	<1	12	4	5	<1
ATAGGGTTTGGCTCCGGGCCTGGCGTCGGTCATCTCTCGC	11232	20211	4793	11387	2333	<1	<1	5	2	7
CACTGGGTTTGGCTCCGGGCCTGGCGTCGGTCGTCTCTCGC	11250	20945	3753	11636	10316	<1	<1	7	2	<1
GCAGGGTTTGGCTCCGGGCCTGGCGTCGGTCGTCTCTCGT	11274	22276	5118	12095	11049	<1	<1	5	2	<1
G— GGGATTGGCTCCGGGCCTGGCGTCGGTCGTCTCTCGC	11295	8671	N	25920	3235	<1	3	N	<1	5
GTAGGGTTTGGCTCCGGGACTGGCGTCGGTCGTCTCTCGC	11312	22889	N	26088	N	<1	<1	N	<1	N
GTAGGGTTTGGCTCCGGGCCTGGCGTCGGTCGTCTCCCCC	11314	22901	N	N	N	<1	<1	N	N	N
-TAGGGTTTGGCTCCGGGCCTGGCGTCGGCCGTCTCTCGC	11323	23256	N	N	N	<1	<1	N	N	N
-TAGGGTTTGGCTCCGGGCCTGGCGTCGGTCATCTCTCGC	11324	23257	N	26468	5114	<1	<1	N	<1	3
GTAGGGTTTGGCTCCGGGCCTGGTGTCGGTCGTCTCTCGC	11385	8755	20732	5584	11530	<1	3	<1	5	<1
GTAGGGTTTGGCTCCTGGCCTGGCGTCGGTCGTCTCTCGC	11397	5175	1916	7535	3274	<1	5	18	4	5
GTAGGGATTGGCTCCGGGCCTGGCGTCGGTCGTCTCTCGC	11465	22881	20709	7531	N	<1	<1	<1	4	N

[a]: Each sequence contains primer regions of TTACGTCAAGGTGTCACTCC and GAAGCATCTCTTTGGCGTG at the 5' end and 3' end, respectively. [b]: N refers to “not detected”. Each point mutation in relation to the top ranked sequence is highlighted in yellow. Each sequence was ranked by the frequency in the DNA pool using a method described in Section A6 above.

Table S3. Ratio paired t-test results comparing the top 15 MSA52 cluster members in WHS, UKS, BZS, SAS and CAS pools as compared to the Round 13 pool. MSA52 cluster member frequencies are not significantly different between the Round 13 reference population and the WHS population. UKS, BZS, SAS and CAS populations of MSA52 are significantly enriched as compared to the Round 13 reference population.

	WHS	UKS	BZS	SAS	CAS
P value	0.7718	<0.0001	<0.0001	<0.0001	<0.0001
Geometric Mean of Ratios	1.053	2.209	3.435	2.877	2.847
SD of log(ratio)	0.2912	0.2467	0.3188	0.3005	0.2961
95% CI	0.7260 to 1.526	1.613 to 3.026	2.288 to 5.159	1.961 to 4.220	1.951 to 4.153

Table S4. All the synthetic oligonucleotides used in this study. Sequences are written 5'-3'. Abbreviations include: 40 bases random region (N_{40} in bold), S, non-amplifiable spacer.

**Selection**		
DNA library (79 nt)		TTACGTCAAG GTGTCACTCC-**N_{40}**-GAAGCATCTC TTTGGCGTG
Forward primer FP1 (20 nt)		TTACGTCAAG GTGTCACTCC
Forward primer FP2 (20 nt)		FAM-TTACGTCAAG GTGTCACTCC
Reverse primer RP1 (19 nt)		CACGCCAAAG AGATGCTTC
Reverse primer RP2 (39 nt)		TTTTTTTTTT TTTTTTTTTT-S-CACGCCAAA GAGAT GCTTC
**Aptamers**		
**Name**	**Size (nt)**	
MSA1	79	TTACGTCAAG GTGTCACTCC CACTTTCCGG TTAATTTATG CTCTACCCGT CCACCTACCG GAAGCATCTC TTTGGCGTG
MSA5	79	TTACGTCAAG GTGTCACTCC ACGGGTTTGG CGTCGGGCCT GGCGGGGGGA TAGTGCGGTG GAAGCATCTC TTTGGCGTG
Mutant Control (MC)	79	TCTTTCGTGC CATCCTGCGG GTGGCTGTCC GAGGCTGGTG GCTCTGCAAG TGCCACGCTT TATCTGAGGT TCGCCGGTA
MSA52	79	TTACGTCAAG GTGTCACTCC GTAGGGTTTG GCTCCGGGCC TGGCGTCGGT CGTCTCTCGC GAAGCATCTC TTTGGCGTG
MSA52-T1	68	TTACGTCAAG GTGTCATTG GCTCCGGGCC TGGCGTCGGT CGTCTCTCGC GAAGCATCTC TTTGGCGTG
MSA52-T2	70	TTACGTCAAG GTGTCACTCC GTAGGGTTTG CTGGCGTCGG TCGTCTCTCG CGAAGCATCT CTTTGGCGTG
MSA52-T3	58	TTACGTCAAG GTGTCACTCC GTAGGGTTTG GCTCCGGGCC TGGCATCTCT TTGGCGTG
MSA52-T4	72	TTACGTCAAG GTGTCACTCC GTAGGGTTTG GCTCCGGGCC TGGCGTCGGT CGCGAAGCAT CTCTTTGGCG TG
MSA52-T5	69	ACGCCAAGGT GTCACTCCGT AGGGTTTGGC TCCGGGCCTG GCGTCGGTCG CGAAGCATCT CCTTGGCGT
CoV2-RBD-1	76	ATCCAGAGTG ACGCAGCACC GACCTTGTGC TTTGGGAGTG CTGGTCCAAG GGCGTTAATG GACACGGTGG CTTAGT
Aptamer-1	76	ATCCAGAGTG ACGCAGCATC GAGTGGCTTG TTTGTAATGT AGGGTTCCGG TCGTGGGTTG GACACGGTGG CTTAGT
CoV2-2	76	ATCCAGAGTG ACGCAGCAGG GATGGGCTCC GGGCTACTGG CGAGGCTTCG GAACAACCGG ACACGGTGGC TTAGTA
S1P	80	GTCTTGACTA GTTACGCCTG GGAGGATTCG GCGCATGGGG ACGGGGGTGG CCCCCCCCCC TCTCATTCAG TTGGCGCCTC
nCoV-S1-A1	80	AGCAGCACAG AGGTCAGATG CCGCAGGCAG CTGCCATTAG TCTCTATCCG TGACGGTATG CCTATGCGTG CTACCGTGAA
SARS2-AR10	45	CCCGACCAGC CACCATCAGC AACTCTTCCG CGTCCATCCC TGCTG

B-MSA1	84	Bio- TTTTTTTACG TCAAGGTGTC ACTCCCACTT TCCGGTTAAT TTATGCTCTA CCCGTCCACC TACCGGAAGC ATCTCTTTGG CGTG
B-MSA52	84	Bio-TTTTTTTACG TCAAGGTGTC ACTCCGTAGG GTTTGGCTCC GGGCCTGGCG TCGGTCGTCT CTCGCGAAGC ATCTCTTTGG CGTG
B-S1P	85	Bio-TTTTTGTCTT GACTAGTTAC GCCTGGGAGG ATTCGGCGCA TGGGGACGGG GGTGGCCCCC CCCCCTCTCA TTCAGTTGGC GCCTC
B-nCoV-S1-A1	85	Bio-TTTTTAGCAG CACAGAGGTC AGATGCCGCA GGCAGCTGCC ATTAGTCTCT ATCCGTGACG GTATGCCTAT GCGTGCTACC GTGAA
B-SARS2-AR10	50	Bio-TTTTTCCCGA CCAGCCACCA TCAGCAACTC TTCCGCGTCC ATCCCTGCTG
B-A25	25	Bio-AAAAAAAAAA AAAAAAAAAA AAAAA

SECTION C: SUPPORTING FIGURES

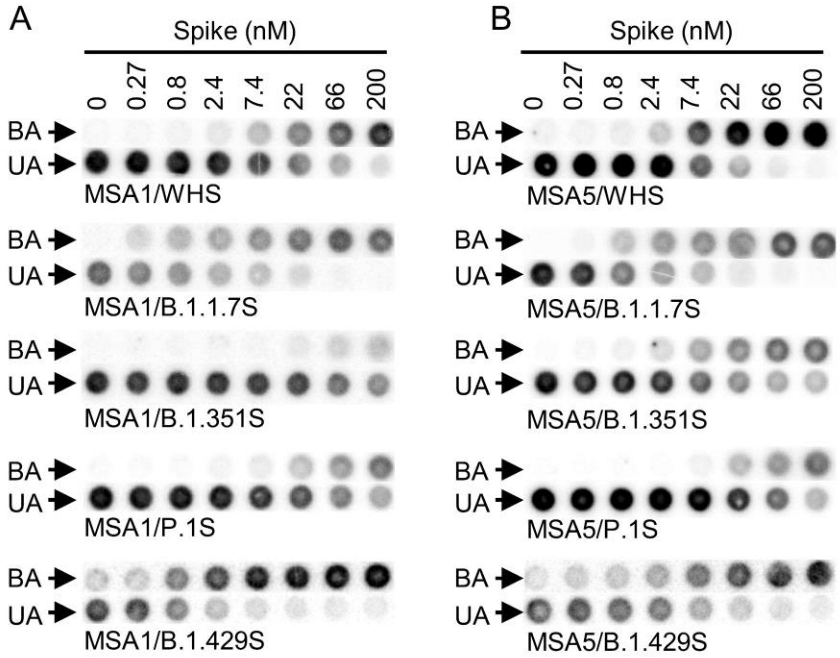

Figure S1. The dot blot results of (A) MSA1 and (B) MSA5 for binding to the full spike proteins of the wild-type Wuhan (WHS) and variants of SARS-CoV-2. BA and UA: bound and unbound aptamers.

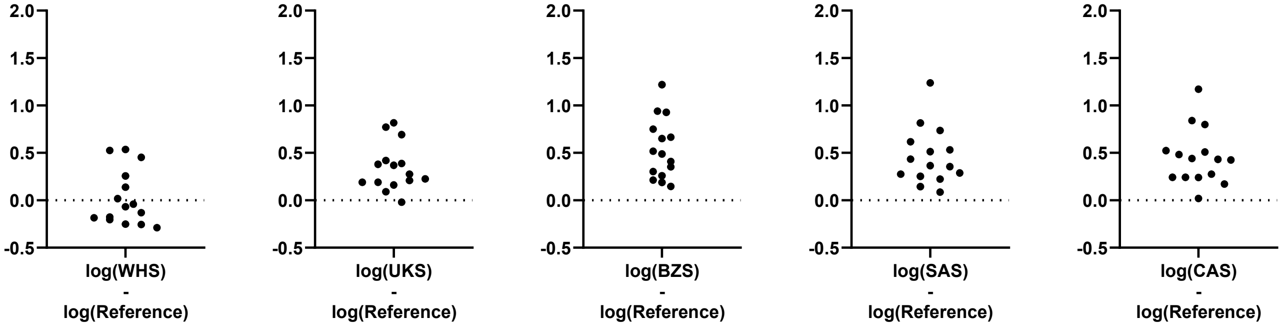

Figure S2. Log(frequency) difference plot of the top 15 MSA52 cluster members in WHS, UKS, BZS, SAS and CAS pools as compared to the Round 13 pool.

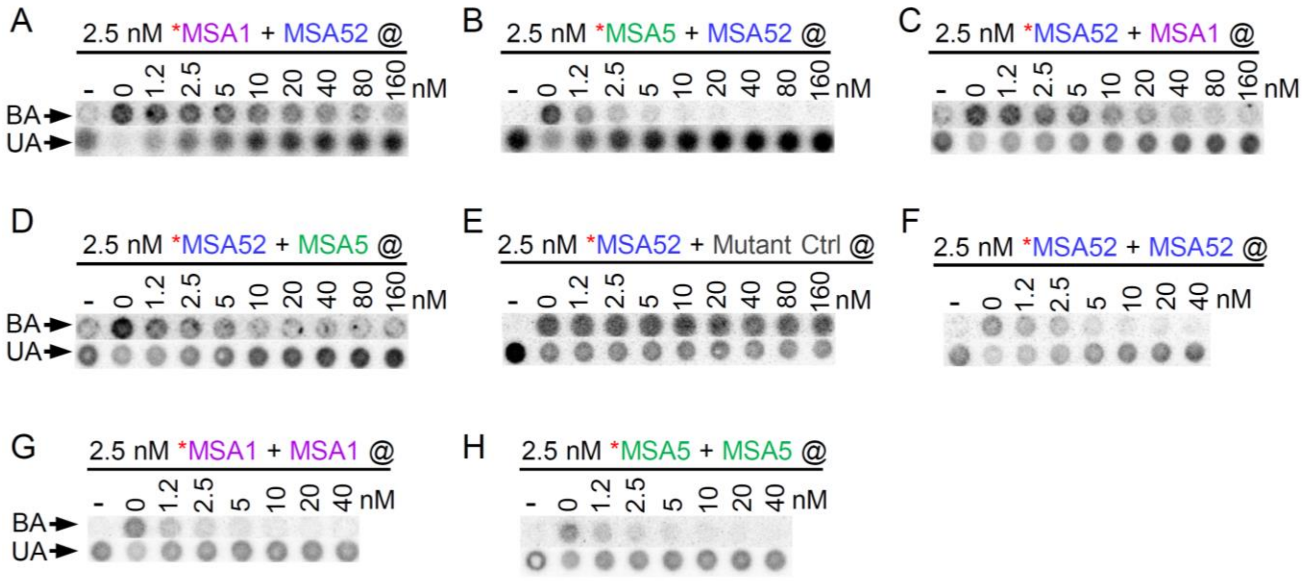

Figure S3. The dot blot results for competition assays: (A) MSA1 and (B) MSA5 competed by MSA52; MSA52 competed by (C) MSA1 and (D) MSA5; (E) a negative control of MSA52 competed by a mutant sequence (shuffled MSA52); self-competitions of (F) MSA52, (G) MSA1 and (H) MSA5. The competed aptamers were at 2.5 nM and radiolabelled (with red asterisk); the competing aptamers were unlabelled and had concentrations of 1.2 - 160 nM. 5 nM of S1 proteins of SARS-CoV-2 were used in the assays. BA and UA: bound and unboud aptamers.

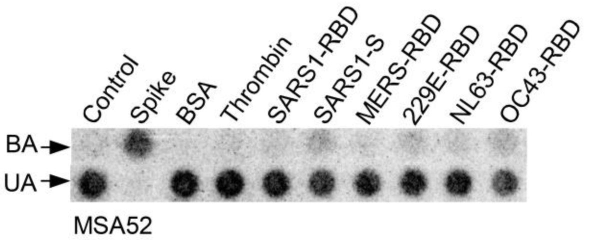

Figure S4. Specificity tests of MSA52 binding to the spike protein of SARS-CoV-2 and control proteins including BSA, human-α-thrombin, and the RBD and spike (S) proteins of SARS-CoV1, RBD proteins of MERS and three seasonal coronavirus 229E, NL63, OC43. 50 nM proteins were in the assays.

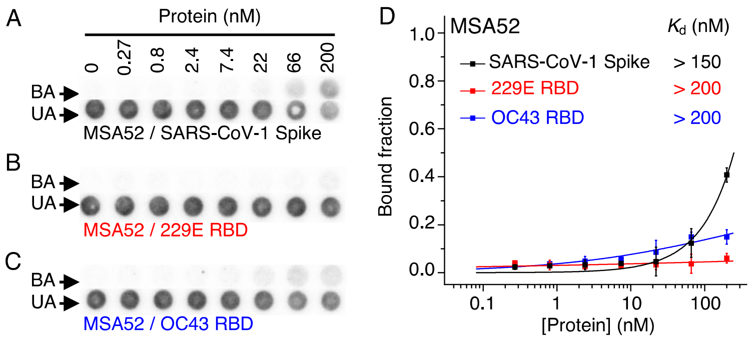

Figure S5. Dot blot results of MSA52 binding to (A) spike protein of SARS-CoV-1, RBD protein of seasonal coronavirus (B) 229E and (C) OC43. (D) Affinity (K_d) determinations. BA: bound aptamer. UA: unbound aptamer.

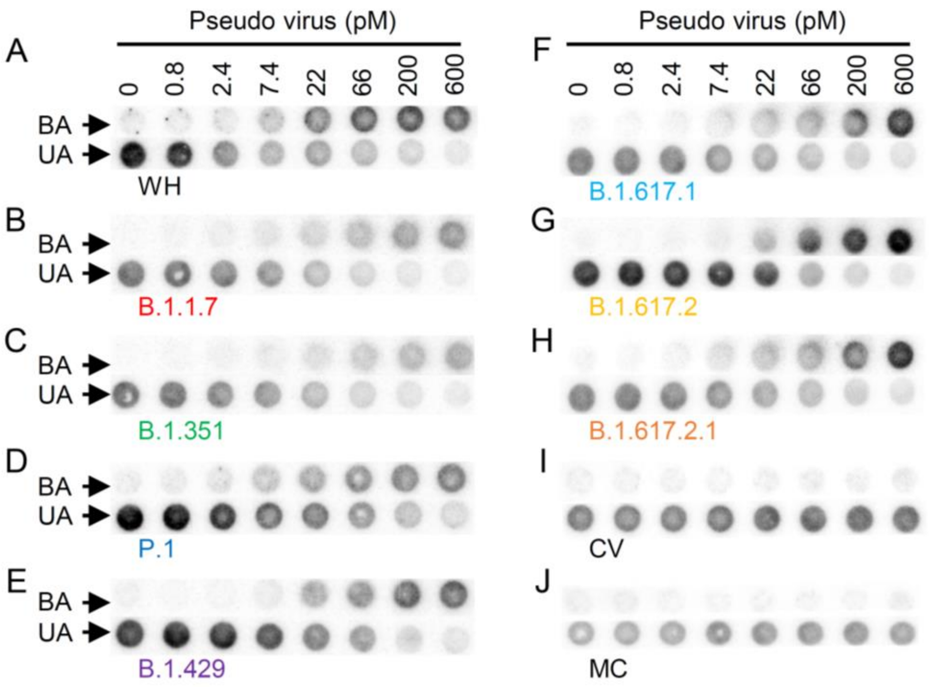

Figure S6. The dot blot results of MSA52 binding to the pseudotyped vlentiviruses of (A) the wild-type (WH) and (B-H) the variants of SARS-CoV-2. (I) MSA52 with control lentiviruses (CV). (J) Inactive mutant sequence of MSA52 (MC) with WH. BA and UA: bound and unboud aptamers.

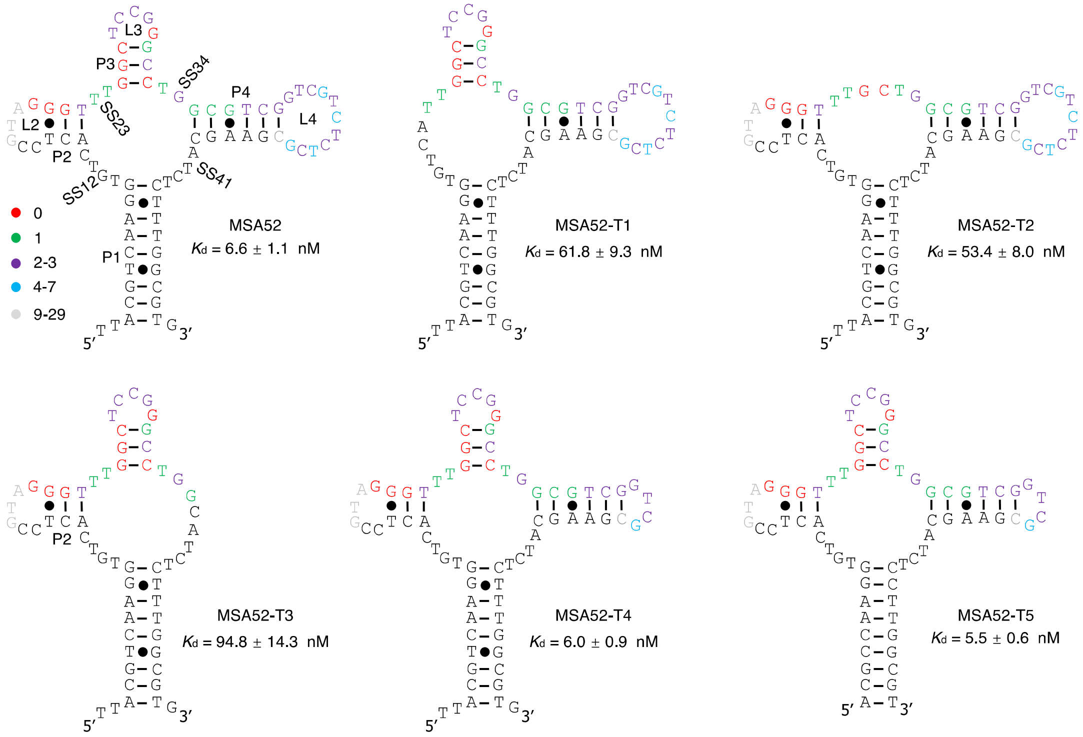

Figure S7. Examination of the predicted secondary structure of MSA52 via sequence truncation. Five truncation mutants (named MSA52-T1 to MSA52-T5). K_d values represent the binding activity for SARS-CoV-2 S1 protein. The nucleotides in the original random-sequence domain are color-coded based on the sequence alignment of the top 100 members of MSA52 family: red – absolutely conserved (0 mutation observed at this poisition within the top 100 sequences); green – highly conserved (1 mutation only); purple – somewhat conserved (2-3 mutations); light blue – less conserved (4-7 mutations); grey – least conserved (9-29 mutations).

References

[1] D. Stadlbauer, F. Amanat, V. Chromikova, K. Jiang, S. Strohmeier, G. A. Arunkumar, J. Tan, D. Bhavsar, C. Capuano, E. Kirkpatrick, P. Meade, R. N. Brito, C. Teo, M. McMahon, V. Simon, F. Krammer, *Curr. Protoc. Microbiol.* **2020**, *57*, e100.

[2] F. Amanat, D. Stadlbauer, S. Strohmeier, T. H. O. Nguyen, V. Chromikova, M. McMahon, K. Jiang, G. A. Arunkumar, D. Jurczyszak, J. Polanco, M. Bermudez-Gonzalez, G. Kleiner, T. Aydillo, L. Miorin, D. S. Fierer, L. A. Lugo, E. M. Kojic, J. Stoever, S. T. H. Liu, C. Cunningham-Rundles, P. L. Felgner, T. Moran, A. García-Sastre, D. Caplivski, A. C. Cheng, K. Kedzierska, O. Vapalahti, J. M. Hepojoki, V. Simon, F. Krammer, *Nat. Med.* **2020**, *26*, 1033–1036.

[3] K. H. D. Crawford, R. Eguia, A. S. Dingens, A. N. Loes, K. D. Malone, C. R. Wolf, H. Y. Chu, M. A. Tortorici, D. Veesler, M. Murphy, D. Pettie, N. P. King, A. B. Balazs, J. D. Bloom, *Viruses* **2020**, *12*, 513.

[4] J. Li, Z. Zhang, J. Gu, H. D. Stacey, J. C. Ang, A. Capretta, C. D. M. Filipe, K. L. Mossman, C. Balion, B. J. Salena, D. Yamamura, L. Soleymani, M. S. Miller, J. D. Brennan, Y. Li, *Nucleic Acids Res.* **2021**, *49*, 7267–7279.

[5] M. Martin, *EMBnet.journal* **2011**, *17*, 10–12.

[6] R. C. Edgar, *Bioinformatics* **2010**, *26*, 2460–2461.

[7] R. C. Edgar, *BMC Bioinformatics* **2004**, *5*, 113.

[8] G. E. Crooks, G. Hon, J.-M. Chandonia, S. E. Brenner, *Genome Res.* **2004**, *14*, 1188–1190.

[9] C. A. Schneider, W. S. Rasband, K. W. Eliceiri, *Nat. Methods* **2012**, *9*, 671–675.

[10] Y. Song, J. Song, X. Wei, M. Huang, M. Sun, L. Zhu, B. Lin, H. Shen, Z. Zhu, C. Yang, *Anal. Chem.* **2020**, *92*, 9895–9900.

[11] M. Sun, S. Liu, X. Wei, S. Wan, M. Huang, T. Song, Y. Lu, X. Weng, Z. Lin, H. Chen, Y. Song, C. Yang, *Angew. Chem. Int. Ed.* **2021**, *60*, 10266–10272; *Angew. Chem.* **2021**, *133*, 10354-10360.

[12] X. Liu, Y. Wang, J. Wu, J. Qi, Z. Zeng, Q. Wan, Z. Chen, P. Manandhar, V. S. Cavener, N. R. Boyle, X. Fu, E. Salazar, S. V. Kuchipudi, V. Kapur, X. Zhang, M. Umetani, M. Sen, R. C. Willson, S. Chen, Y. Zu, *Angew. Chem. Int. Ed.* **2021**, *60*, 10273–10278; *Angew. Chem.* **2021**, *133*, 10361-10366.

[13] A. Gupta, A. Anand, N. Jain, S. Goswami, A. Anantharaj, S. Patil, R. Singh, A. Kumar, T. Shrivastava, S. Bhatnagar, G. R. Medigeshi, T. K. Sharma, *Mol. Ther. - Nucleic Acids* **2021**, *26*, 321–332.

[14] G. Yang, Z. Li, I. Mohammed, L. Zhao, W. Wei, H. Xiao, W. Guo, Y. Zhao, F. Qu, Y. Huang, *Signal Transduct. Target. Ther.* **2021**, *6*, 1–4.

[15] A. S. Peinetti, R. J. Lake, W. Cong, L. Cooper, Y. Wu, Y. Ma, G. T. Pawel, M. E. Toimil-Molares, C. Trautmann, L. Rong, B. Mariñas, O. Azzaroni, Y. Lu, *Sci. Adv.* **2021**, *7*, eabh2848.
